# Amantadine intoxication despite moderate renal dysfunction: A case of combined use with donepezil

**DOI:** 10.1002/ccr3.2803

**Published:** 2020-03-16

**Authors:** Kouji Okada, Takashi Uno, Miho Utsumi, Kensuke Usui, Masashi Nakamura, Ichiro Nakashima, Eiji Suzuki, Yoshiteru Watanabe

**Affiliations:** ^1^ Department of Clinical Pharmaceutics and Pharmacy Practice Tohoku Medical and Pharmaceutical University Sendai Japan; ^2^ Department of Pharmacy Tohoku Medical and Pharmaceutical University Hospital Sendai Japan; ^3^ Division of Neurology Tohoku Medical and Pharmaceutical University Hospital Sendai Japan; ^4^ Division of Psychiatry Tohoku Medical and Pharmaceutical University Sendai Japan

**Keywords:** amantadine, cerebral infarction, donepezil, intoxication, involuntary movement, moderate renal dysfunction

## Abstract

Amantadine intoxication occurred despite moderate renal dysfunction. This may have been affected by the use of donepezil, and we require careful attention to these combinations.

## Graphical abstract

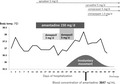

## 1 INTRODUCTION

Administration of amantadine and donepezil to improve patient with Alzheimer‐type dementia and cerebral infarction led to involuntary movements. Amantadine blood concentration reached a toxic level (3647 ng/mL). It is necessary to carefully monitor patients with even moderate renal dysfunction, who receive combinations of amantadine and drugs, such as donepezil.

Amantadine acts on dopaminergic neurons and is used to treat symptoms of Parkinson's disease.^1^ This drug also alleviates the decrease in spontaneity due to late effects of cerebral infarction,^2^ and it is approved for this purpose in Japan. As elimination is primarily achieved through renal clearance, half‐life of amantadine is longer in patients with renal dysfunction.^3^ The renal clearance of amantadine can exceed creatinine clearance (Ccr), suggesting that tubular secretion can play a role in elimination. Hence, amantadine doses must be reduced in patients with renal dysfunction to avoid toxicity.^4^ So far, amantadine intoxication has only been reported in patients with renal failure.^5^, ^6^, ^7^ However, the aim of this study is to report our clinical experience with a patient who developed toxic symptoms due to an increase in blood amantadine concentration during the combined use of donepezil, while being diagnosed with cerebral infarction and moderate renal dysfunction.

## 2 CASE HISTORY

The patient was an 80‐year‐old woman. Her medical history consisted of arrhythmia that had developed 16 years ago, Alzheimer's disease 4 years ago, and a fracture in the right femoral neck 2 years ago. Due to motor aphasia and right hemiparesis, she was admitted to our hospital and diagnosed with cerebral infarction. Findings upon admission included height 152.0 cm, weight 52.2 kg, blood pressure 149/59 mm Hg, pulse 89 beats per minute, temperature 36.5°C, and SpO_2_ 93%‐95% (room air). Medicines that were brought were canceled on admission.

**Figure 1:**
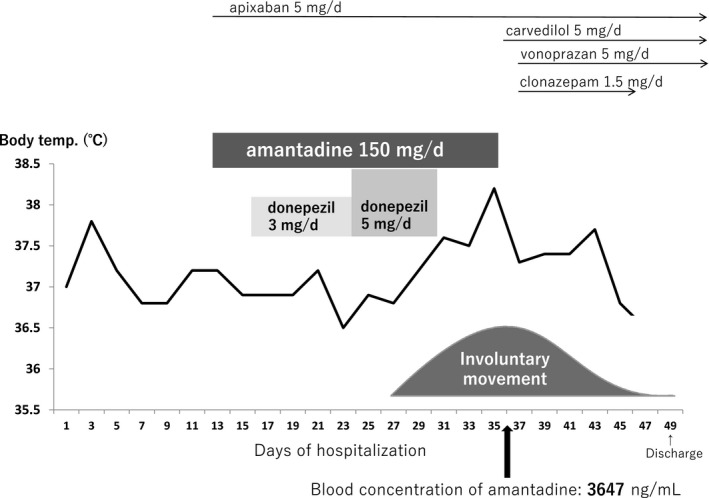
Time course of drug administered and symptoms

The time course of drug administration and symptoms are illustrated in Figure 1, and the transition of laboratory values and vital signs are summarized in Table 1. On the day after hospitalization, the patient received heparin sodium, edaravone, and fluid replacements. On the 13th day, right hemiparesis was still persistent. In addition to hemiparesis, her decreased spontaneity due to the late effects of cerebral infarction diminished her oral intake. Therefore, she received simultaneous administration of amantadine (150 mg/d for the loss of spontaneity with the late effects of cerebral infarction) and apixaban via a nasogastric tube. Furthermore, because there was not much improvement in her spontaneity and mobility due to Alzheimer's dementia, 3 mg/d of donepezil was administered on the 16th day, followed by 5 mg/d donepezil on the 24th day. On the 27th day, involuntary movements (stiffening and tremor) started appearing. On the 30th day, tremors were observed across the entire body when the patient was being addressed or touched, which were presumed to be Gegenhalten. We also suspected that the tremors were extrapyramidal symptoms and, hence, stopped the administration of donepezil. On the 34th day, as fever and tachycardia had developed, chest X‐ray and blood tests were performed; no infections, such as pneumonia, were found. On the 35th day, involuntary movements and fever increased, which we suspected to be due to amantadine intoxication. Hence, the administration of amantadine was discontinued. On the 36th day, we added carvedilol for the treatment of tachycardia. Blood concentration of amantadine was measured, which was found to be at a toxic level (3647 ng/mL). Creatine kinase was within the normal range, and no malignant syndromes were found. On the 37th day, clonazepam was initiated due to the persistence of the myoclonus‐like involuntary movement. On the 40th day, both stimulated systemic hypertonia and involuntary movement seemed to improve. On the 49th day, the toxic symptoms disappeared and the patient was discharged from the hospital.

**Table 1:** Summary of laboratory values and vital signs from day 1‐day 47, where “day” refers to time after hospitalization

		Day 1	Day 13	Day 27	Day 36	Day 47
Laboratory data						
WBC	×10^3^/µL	5.8	4.6	5.1	7.8	4.4
Hgb	g/dL	15.3	13.9	13.1	11.7	12.2
PLT	×10^4^/µL	16.2	29.1	27.8	24.4	38.2
AST	U/L	12	9	20	16	17
ALT	U/L	4	5	40	22	23
LDH	U/L	150	210	256	235	218
CK	U/L	15	–	–	47	–
Scr	mg/dL	0.81	0.61	0.65	0.65	0.56
BUN	mg/dL	13	21	20	30	20
Ccr	mL/min	43	58	54	54	63
Alb	g/dL	3.9	2.9	3.1	3.2	2.6
Na	mEq/L	137	–	132	132	138
K	mEq/L	4.6	–	4.5	4.0	4.5
Cl	mEq/L	100	–	96	96	105
CRP	mg/dL	0.03	1.47	0.93	1.65	1.22
Vital sign						
SpO2	%	93‐98	95	98	85‐96	96‐97
PR	bpm	89‐105	85‐90	55	110‐133	100
SBP	mm Hg	132‐149	114‐136	118	128‐130	125
DBP	mm Hg	59‐88	60‐73	64	66‐84	68

Note

“day” means days of after hospitalization.

Abbreviations: Alb, albumin; ALT, alanine aminotransferase; AST, aspartate aminotransferase; BUN, blood urea nitrogen; Ccr, creatinine clearance; CK, creatine kinase; Cl, serum chloride; CRP, C‐reactive protein; DBP, diastolic blood pressure; Hgb, haemoglobin; K, serum potassium; LDH, lactate dehydrogenase; Na, serum sodium; PLT, platelet count; PR, pulse rate; SBP, systolic blood pressure; Scr, serum creatinine; SpO_2_, blood oxygen saturation; WBC, white blood cell count.

## 3 DISCUSSION

We experienced a case of amantadine intoxication despite the presence of only moderate renal dysfunction. Previous reports indicate that toxic symptoms are observed if amantadine blood concentration exceeds 3000 ng/mL.^8^ In our case, we found that amantadine had reached toxic levels. We suspect that individual differences in the ability to excrete the drug and/or the effects of combined use of donepezil caused amantadine intoxication in this case.

Nishikawa et al (2009) also reported that plasma amantadine concentration exhibited a significant negative correlation with Ccr. And a 4‐fold range in blood concentration was noted when 100 mg/d of amantadine was administered to patients with normal renal function.^8^ The dosage of amantadine in the package insert for the late effect of cerebral infarction was "100‐150 mg/d divided into 2‐3 doses." The dosage of amantadine was within the standard dose range; however, it may have been an overdose for our patient. Other reports also describe the use of amantadine intoxication to improve the spontaneity of patients with moderate renal dysfunction.^9^ As most of amantadine is excreted into urine in the unchanged form, individual differences in the ability to excrete the drug are the cause of fluctuation in the blood amantadine levels. A report described how amantadine intoxication at 150 mg/d in a patient with moderate renal dysfunction could be controlled by the re‐administration of amantadine at 50 mg/d initially and then gradually increasing the dosage.^9^

The potency of the drug interaction between amantadine and donepezil should be studied, as amantadine intoxication symptoms were found to occur while using donepezil. Donepezil is effective in reducing involuntary movements due to Alzheimer's dementia,^10^ which was also observed in this case. Amantadine is known as a drug having a large ratio of tubular secretion to the renal clearance (from the information in the package insert). Memantine, an *N*‐methyl‐d‐aspartate receptor inhibitor with a structure similar to amantadine, is a substrate of the organic cation transporter 2 (OCT2) and the multidrug and toxin extrusion protein 1 (MATE1).^11^ OCT2 is primarily a renal uptake transporter that is expressed on the basolateral (blood) side of the proximal tubule cells. MATE1 is an apically expressed poly‐specific proton antiporter that mediates renal efflux of diverse substrates that are primarily organic cations. Donepezil inhibits both OCT2^12^ and MATE1.^13^ We hypothesize that the combined use of amantadine and donepezil led to competition in transport of amantadine through OCT2 and MATE1 leading to inhibition of its excretion and a consequent increase in its blood concentration. Although pharmacokinetics data are not available for the combination of amantadine and donepezil, there are several reports on memantine, which has a structure and activity similar to those of amantadine. An in vivo study reported that the combination of donepezil and memantine decreased clearance of memantine and hence increased its blood concentration and its area under the curve.^14^ However, no significant pharmacokinetic interaction was observed in a co‐administration study of donepezil and memantine conducted on healthy human subjects.^15^ Moreover, meta‐analyses have also investigated the combined efficacy and safety of donepezil and memantine for Alzheimer‐type dementia.^16^ Currently, the effect of the combination of memantine and donepezil is not considered a clinical problem. However, to the best of our knowledge, there are no reports on the direct evaluation of the effects of the combination of amantadine and donepezil. Drug interactions related to renal transporters are becoming clear, but there are many obscurity points. And these drug interactions are still not well recognized in clinical practice.^17^ In fact, the package insert does not contain any information about drug interactions between amantadine and donepezil. In this report, we presume amantadine intoxication because of their interaction through renal transporter inhibition although the mode of action will need further investigation.

## 4 CONCLUSIONS

Amantadine is excreted renally at a high rate in the form of the unchanged drug. In this case, amantadine concentrations in the blood increased to toxic levels despite moderate renal dysfunction. It is necessary to carefully monitor the patient for toxic symptoms caused by amantadine, especially in patients receiving a combination of amantadine and drugs such as donepezil.

## CONFLICT OF INTEREST

We declare no conflict of interest.

## AUTHOR CONTRIBUTIONS

KO: designed the study, analyzed and interpretation of data, and wrote the manuscript. TU and MU: provided pharmaceutical care to patient, collected data, and were involved in manuscript drafting and editing. KU: contributed to case interpretation and was involved in manuscript drafting and editing. MN and IN: provided clinical care to patient and were involved in manuscript drafting and editing. ES and YW: were involved in manuscript drafting and editing and contributed to supervision. All authors approved the final version of the manuscript and agreed to be accountable for all aspects of the work in ensuring that questions related to the accuracy or integrity of any part of the work are appropriately investigated and resolved.
